# Severe combined immunodeficiency: a case series from a paediatric hospital in Kenya

**DOI:** 10.11604/pamj.2021.39.56.26419

**Published:** 2021-05-20

**Authors:** Rohini Kalagouda Patil, Anne Irungu, Beatrice Kabera, Doreen Karimi Mutua, Wangui Manguyu, David Kiptum Lagat, Kaumbulu Cleopas Mutua

**Affiliations:** 1 Department of Paediatric Critical Care, Gertrude´s Children´s Hospital, Nairobi, Kenya,; 2 Department of Paediatric Pulmonology, Gertrude´s Children´s Hospital, Nairobi, Kenya,; 3 Department of Clinical Pathology, Gertrude´s Children´s Hospital, Nairobi, Kenya,; 4 Department of Hematology/Oncology, Gertrude´s Children´s Hospital, Nairobi, Kenya,; 5 Department of Paediatrics, Gertrude´s Children´s Hospital, Nairobi, Kenya,; 6 Department of Paediatrics, University of Nairobi, Nairobi, Kenya

**Keywords:** Severe combined immunodeficiency, primary immunodeficiency disorders, children, Kenya

## Abstract

Severe Combined Immunodeficiency (SCID) involves the lymphocyte lineage and mimics Human Immunodeficiency Virus (HIV) disease common in our region, making it difficult to diagnose and manage effectively. SCID in East Africa stands underdiagnosed because of lack of awareness and diagnostic resources. A case series of three SCID patients admitted to a Tertiary Paediatric Centre in Kenya between 2016 and 2019. The clinical presentations, laboratory findings, management and outcome for each were studied. Three cases were diagnosed between the ages of 4 to 15 months. Two of them were male and one was a female. All had a history of previous sibling death. There was no parental consanguinity. All presented with pneumonia. One of them had vaccine acquired Rotavirus infection and a persistent generalised maculopapular rash. The T, B cell profile was T- B- in two and T- B+ in one case and the immunoglobulins were reduced in all. All the cases were fatal. Thus, Primary immunodeficiency disorders are prevalent in East Africa. A proper clinical history, examination and laboratory tests like a haemogram, peripheral blood film can aid to suspect and diagnose SCID even with limited resources.

## Introduction

Primary immunodeficiency disorders (PID) comprises of genetically heterogeneous group of disorders caused by inborn defects in components of the immune system. Apart from causing unusual infections or typical infections with unusually long duration and severe course, the poorly regulated immune system in PID patients also causes inflammation, autoimmunity and malignancy [1]. Severe Combined Immunodeficiency (SCID) is the prototype of PID. Severe T and B cell deficiency and occasionally Natural Killer (NK) cell deficiency leads to serious life threatening infections. Children with SCID seek medical attention early in life and commonly present with failure to thrive, pneumonia, sepsis, chronic diarrhoea and persistent oral candidiasis together with cutaneous manifestations including bacterial, fungal, and viral skin infections, erythroderma, or eczematous rash [2]. However, because of their defective immune responses, these patients are often relatively less symptomatic for any given degree of infection initially, but symptoms may worsen with each infection. Infants with unrecognized SCID are susceptible to live vaccine-acquired diseases such as Bacillus Calmette-Guerin (BCG), Varicella and Rotavirus vaccines [3]. Without intervention, they usually result in severe infection and death by the age of 2 years.

Severe Combined Immunodeficiency (SCID) immunophenotypes can be classified according to the presence (T-B+ SCID) or absence (T-B- SCID) of B cells in the peripheral blood. Both main groups of SCID include forms with or without NK cells. X-linked (XL) and autosomal recessive (AR) forms of SCID have been identified, with the XL-SCID as the most common worldwide form (~50%) of SCID in human subjects [4]. However, with high consanguinity, nearly 60% in North Africa and sub-Saharan Africa, it allows the emergence of many autosomal recessive (AR) forms of SCID [5]. Primary immunodeficiency disorders in the African continent, mainly East Africa stand underdiagnosed because of lack of awareness and diagnostic resources [6]. The diagnosis of PID is limited by high prevalence of paediatric infectious diseases like Human Immunodeficiency Virus (HIV) together with malnutrition in this region and thus the low index of suspicion, which may explain the paucity of case reports on PID in Africa, most coming from Northern and Southern Africa [4-6]. The primary treatment of choice for most types of SCID is Allogeneic Hematopoietic Stem Cell Transplant (HSCT). Infants undergoing transplantation in the first 3.5 months of life have a higher rate of survival [7]. We present a case series of three SCID patients with an objective to increase awareness of the disease in our region and help clinicians diagnose and plan their resources well. Early detection and intervention before infections set in is the ideal goal for children with SCID.

## Methods

**Setting:** Gertrude´s Children´s Hospital is a Private Paediatric Quaternary Centre providing care in general paediatrics and various paediatric subspecialties. It´s based in the capital city of Kenya, Nairobi on the North Eastern area and receives referrals from both Public and Private Healthcare facilities and provides services to children coming from all over Kenya, East and Central Africa. The hospital attends to over 350,000 children as outpatient each year and admits more than 10,000 children as inpatient each year.

**Study population:** children from birth to 18 years admitted as inpatient and had a confirmed diagnosis of SCID in the database were included in the case series. SCID diagnosis was based on clinical parameters of any PID case in the database that fitted the 2017 IUIS Phenotypic Classification for Primary Immunodeficiencies [8].

**Design:** we performed a retrospective audit of our hospital database and three cases with SCID were found within a period of 3.5 years, between 2016 when we diagnosed our first case up to 2019. The hospital Database mainly includes clinical, laboratory and radiological information about the patients.

**Data collection:** patient biodata, History, clinical features and laboratory data was assessed. Enumeration of selected peripheral-blood leukocytes, including CD3+ T cells, CD4+ T cells, CD 19/20+ B cells, and CD 16/56+ natural killer cells, was performed by means of flow cytometric analysis. Immune profiles were determined by Flow cytometry (BD FACSCalibur, BD diagnostics) at a Referral Laboratory in India. Humoral immune responses were measured by determination of the serum IgG, IgA, IgM levels.

**Ethical considerations:** we obtained consent from the Hospital Head of Clinical Services to publish the data and have maintained patient anonymity.

## Results

The mean age was 7 months with a range of 4-10 months. All patients had received their BCG vaccination at birth as per our national expanded programme on immunization. All of the parents and patients were ethnic Africans and no 1^st^ or 2^nd^ degree consanguinity was noted. Tests for Human immunodeficiency virus (HIV) and GeneXpert® for Mycobacterium tuberculosis were negative for all of them. All cases were admitted in the Intensive Care Unit and succumbed before definitive treatment of their disorder was instituted. The following is the detailed description of each case.

**Case 1:** an 8 month old male child presented with pneumonia, irritability and seizures that was not improving on treatment for more than a week at the referring hospital. He also showed features of congestive cardiac failure and failure to thrive. He had previously been admitted and treated for pneumonia at 7 months of age at a different facility. There was a male and a female sibling death in early childhood from severe pneumonia. Chest X-ray showed features of pneumonia and a 2D echocardiography showed dilated cardiomyopathy. The important laboratory tests are summarised in Table 1. He was diagnosed to have T -B- SCID with low immunoglobulin levels at 10 months of age and received a dose of intravenous immunoglobulin together with appropriate antibiotics and prophylactic antiviral and antifungal medications but died within few days of diagnosis.

**Table 1:** immunological profiles of the patients

Case	T, B-cell Profile	White cell count (x10^3^/l)	Absolute lymphocyte count (x10^3^/l)	T-cell Subsets	B-cell Subsets	Natural killer (NK) cells	Immunoglobulin levels (IU/mL)
				CD3 cells/μL	CD 19 cells/μL	CD(16+56) cells/μL	
1	T^-^B^-^	4.72	0.31	118 (1900-5900)	63 (610-2600)	11 (50-1160)	Low
2	T^-^B^-^	14.34	1.21	114 (1900-5900)	567 (610-2600)	Not done	Low
3	T^-^B^+^	6.28	1.13	36 (2500-5600)	849 (74-441)	156 ( 170- 830)	Low

CD: cluster of differentiation; IU: international units

**Case 2:** a 7 month old female presented with severe pneumonia which progressed to acute respiratory distress syndrome (ARDS) with septic shock. She was previously treated for pneumonia at 2 months of age at a different facility. She also had severe gastroesophageal reflux disease, atopy and failure to thrive. Two of her siblings died at early infancy from severe infections and two others were alive and well. There was a history of atopy and allergy on the paternal side. Her chest X-ray revealed features of ARDS and she received treatment for the same. She received a dose of intravenous immunoglobulins (IVIG) and appropriate antimicrobials. Her laboratory parameters are summarised in Table 1. She also had T -B- SCID with low immunoglobulin levels. She was diagnosed within a month of admission but succumbed soon after.

**Case 3:** a 4 month old male child presented with severe pneumonia and rotavirus gastroenteritis. He was previously treated for upper respiratory tract infection and gastroesophageal reflux disease as an outpatient case at 3 months of age. History of male sibling death in early childhood from severe pneumonia was present. He also had severe progressive dermatitis and failure to thrive. He suffered from persistent rotavirus infection which may have been a vaccine-acquired disease and later developed severe sepsis. The Chest X-ray revealed pneumonia and a 2D echocardiogram was normal. The pneumonia was treated with appropriate antibiotics. He was diagnosed to have T -B+ SCID with low immunoglobulin levels at the age of 8 months. His laboratory findings are as per Table 1, further genetic studies were planned and he also got human leucocyte antigen (HLA) typing for a matched donor for Hematopoietic stem cell transplantation (HSCT) abroad. He received regular intravenous immunoglobulins and prophylactic antimicrobials awaiting transplant but died due to overwhelming sepsis at the age of 10 months.

## Discussion

Severe Combined Immunodeficiency (SCID) is a group of genetically inherited diseases resulting from different gene mutations that affects humoral and cellular immunity [8]. The Jeffrey Modell Centres Network on PID did a global survey in 2018 covering 6 continents, 86 countries and 358 institutions. The prevalence of PID cases in Africa reported was 1, 836 patients against a global total of 94,024 [9]. This may not be an accurate reflection of our prevalence as Africa is a highly populated continent with a wide gene pool, thus the prevalence is expected to be much higher. Bousfiha *et al*. in 2013 made an upper estimate of PID cases at 902,631 in Africa in their review of data from different country registries and population based studies [4]. The true prevalence specifically of SCID is unknown in East Africa, no studies were found in a recent systematic review by Erjaee *et al*. [6] and none of the countries have neonatal screening programs or national registries apart from North and South Africa. There is only one case report from Kenya about PID diagnosed from post-mortem tissue samples [10]. To our knowledge, our case series is the first ever report describing SCID from this region. In a retrospective descriptive study on PIDs done in South Africa over a period of 37 years, there were 25 SCID cases among the total 252 cases of PID identified (9.9%) [11]. Combined cellular and humoral deficiency were the third most common PID in South Africa and antibody deficiencies being their most common PID similar to that reported from the western countries [12]. However, Combined Immunodeficiency Disorders (CID) were the most common PIDs with a prevalence of 41, 27, and 19% of total PID cases in the North African countries of Egypt, Tunisia, and Morocco, respectively [13-15].

The initial clue to diagnosis in our cases was persistent lymphopenia, HIV negative, early onset and severe recurrent infections with a sibling death due to severe infection [16]. We had predominantly male cases with only one female affected similar to that seen in South Africa which has a similar ethnic profile of predominantly black Africans as Kenya. Despite the high incidence of consanguinity expected in our region, none of the cases above had a parental consanguinity similar to that seen in South Africa which had only 1.2% [12]. However, North African countries like Tunisia [14], Egypt [13], and Morocco [15] which are predominantly of Arab ethnicity, they had 58.2%, 62.5%, 43.2% parental consanguinity respectively and thus AR inheritance was more common in that region. We were unable to do further genetic studies to specify the inborn immune defect in our cases due to lack of diagnostic resources in the country and thus did not know the type of inheritance that existed in our SCID patients. Even in better resourced centres, Genetic or chromosomal confirmation of PID diagnosis was not often obtained and was reported as 16.7%, 13.8% and 36% in South Africa [12], Tunisia [14], and European countries [17] respectively. The challenge of PID diagnosis in our Country is due to the higher burden by common secondary causes of immunodeficiency like HIV disease with a 4.9% national prevalence and severe malnutrition at a third of the under 5 year olds [17], mimicking the symptoms and lack of a higher index of suspicion. Lack of immunodiagnostic capacity, cost of expatriating samples and no stem cell transplant centres adds to the difficulty of managing the disease. The mean age at diagnosis was 8.6 months in our series, similar to that seen in Egypt but the mean age at diagnosis in Middle-East countries was as early as 5 months probably because of greater awareness in the region [2].

The most common infections were lower respiratory tract infections and also neurological symptoms similar to that reported by Nofech-Mozes *et al*. [18]. One of the cases presented with vaccine-acquired Rotavirus infection similar to that reported by Patel, Niraj C *et al*. [19]. Tuberculosis gene test was negative in all our cases in spite of our region being endemic for Mycobacterium tuberculosis. BCG vaccine-induced disseminated tuberculosis was not seen unlike in other studies [20]. The T, B and NK cell profile on flow cytometry was T- B- NK-, T- B-NK (The NK levels were not done) and T- B+ NK- for each case respectively and the immunoglobulin levels were reduced in all the patients. In comparison, T-B+ SCID were more common in South Africa and T-B- SCID was more common in North Africa [11,15]. All the children died of overwhelming sepsis before receiving definitive treatment for their immune defect. Thirty-six percent (36%) of known deaths in PID in South Africa were due to SCID [11]. Egypt, Morocco and Tunisia had a mortality of 23.4%, 28.8% and 34.5%, respectively for the PID cases, majority being CID [13-15]. The benefit of early diagnosis includes early interventions that may improve prognosis like HSCT, enables counselling of parents with regards to the risk to their subsequent children and better utilisation of resources for managing common PIDs on a national scale, once the prevalence is known.

**Limitations:** apart from being a retrospective study which has its own limitations, we did not get an opportunity to conduct further genetic studies in these patients which would have provided better information that would aid in better counselling of the families for future pregnancies.

## Conclusion

Primary Immunodeficiency Disorders (PIDs) are likely prevalent in East Africa. A thorough patient and family history taking together with investigations like a full haemogram, peripheral blood film to assess for lymphopenia and morphology, HIV status, followed by specific immunological tests like immunoglobulin levels, lymphocyte subsets, B and T cell markers, can give clues to the diagnosis of SCID. High index of suspicion and early diagnosis is the key to improve outcomes. HSCT and Gene therapies for management of SCID are unavailable locally but can be developed as the awareness and diagnosis continues to increase. A registry for future cases and prospective studies to determine the common defects should be instituted nationally.

### What is known about this topic

- Severe combined immunodeficiency in East Africa stands under-diagnosed because of lack of awareness;
- Diagnosis of primary immunodeficiency is challenging in the region because of high prevalence of pediatric infectious diseases like HIV together with malnutrition.

### What this study adds

- Severe combined immunodeficiency is likely prevalent in East Africa;
- The case series helps create awareness by identifying clues in the history, clinical presentation and simple laboratory tests which aid in the early diagnosis;
- To plan the limited resources carefully for diagnosis and definitive management.
